# Identification of mutations that causes glucose-6-phosphate transporter defect in tunisian patients with glycogenosis type 1b

**DOI:** 10.1186/s13098-023-01065-2

**Published:** 2023-04-28

**Authors:** Latifa Chkioua, Yessine Amri, Chayma Sahli, Ferdawes Ben Rhouma, Amel Ben Chehida, Neji Tebib, Taieb Messaoud, Hassen Ben Abdennebi, Sandrine Laradi

**Affiliations:** 1 Research Laboratory of Human Genome and Multifactorial Diseases, Faculty of Pharmacy, University of Monastir, Monastir, Tunisia; 2 Faculty of Pharmacy, University of Monastir, Street Avicenne, Monastir, 5000 Tunisia; 3 Biochemistry Laboratory (LR 00SP03), Bechir Hamza Children’s Hospital, Tunis, Tunisia; 4 Department of Educational Sciences, Higher Institute of Applied Studies in Humanity, University of Jendouba, Le Kef, Tunis, Tunisia; 5 Pediatrics Department, La Rabta Hospital, Tunis, Tunisia; 6 Research Laboratory: LR12SPO2 Investigation and Management of Inherited Metabolic Diseases, Faculty of Medicine of Tunis, Tunis El Manar University, Tunis, Tunisia; 7 The Auvergne-Rhône-Alpes Regional Branch of the French National Blood System EFS/GIMAP, EA 3064, Saint Etienne, 42100 France

**Keywords:** Glycogenosis, Glucose-6-phosphate transporter (SLC37A4), Bioinformatics tool, Mutations

## Abstract

**Background:**

Glycogen storage disease type 1b (GSD1b) is an autosomal recessive lysosomal storage disease caused by defective glucose-6-phosphate transporter encoded by SLC37A4 leading to the accumulation of glycogen in various tissues. The high rate of consanguineous marriages in Tunisian population provides an ideal environment to facilitate the identification of homozygous pathogenic mutations. We aimed to determine the clinical and genetic profiles of patients with GSD1b to evaluate SLC37A4 mutations spectrum in Tunisian patients.

**Methods:**

All exons and flanking intron regions of *SLC37A4* gene were screened by direct sequencing to identify mutations and polymorphisms in three unrelated families with GSD1b. Bioinformatics tools were then used to predict the impacts of identified mutations on the structure and function of protein in order to propose a function-structure relationship of the G6PT1 protein.

**Results:**

Three patients (MT, MB and SI) in Families I, II and III who had the severe phenotype were homoallelic for the two identified mutations: p.R300H (famillies I, II) and p.W393X (Family III), respectively. One of the alterations was a missense mutation p.R300H of exon 6 in *SLC37A4* gene. The analysis of the protein structure flexibility upon p.R300H mutation using DynaMut tool and CABS-flex 2.0 server showed that the reported mutation increase the molecule flexibility of in the cytosol region and would probably lead to significant conformational changes.

**Conclusion:**

This is the first Tunisian report of SLC37A4 mutations identified in Tunisia causing the glycogenosis type Ib disease. Bioinformatics analysis allowed us to establish an approximate structure-function relationship for the G6PT1 protein, thereby providing better genotype/phenotype correlation knowledge.

## Introduction

Glycogen storage disease type I GSD1b) is a group of rare inborn errors of metabolism disorders caused by deficiencies in the activities of glucose-6-phosphatase-α (G6Pase-α)/glucose-6-phosphate transporter (G6PT) complexes. GSD type 1a represents the most frequent type of GSD1, responsible for > 80% of GSD 1 patients [1] while GSD type 1b is estimated to represent ~ 20% of 53 cases [2].

The G6Pase-α and G6PT complexes are functionally coupled as follow: G6PT1 transports G6P from the cellular cytosol into the lumen of the endoplasmic reticulum, where G6P is hydrolyzed to glucose and inorganic phosphate by G6Pase-α enzyme [3]. GSD1b can result of mutations in SLC37A4, which encodes glucose-6-phosphate transporter 1 (G6PT1). SLC37A4 (OMIM #602,671; GenBank NM_001467.6) is located on the short arm of chromosome 11 (11q) and contains 9 exons that are distributed across ~ 5.3 kb of genomic DNA. G6PT codes for 429 amino-acid peptide, predicted to contain ten transmembrane endoplasmic reticulum domains. The C-terminal and N-terminal tails are predicted to be oriented towards the cytoplasm [4, 5]. More than 90 mutations (www.hgmd.org. 2019) have been elucidated and classified as helical mutations, on non helical mutations and on N-terminal and C-terminal domains.

GSD 1b appears in the first year of life associated with the apparition of hypoglycemia. This pathologic condition is characterized by the accumulation of intracytosol G6P, leading to alternative pathways of glucose metabolism such as excessive formation of triglycerides, lactate, and uric acid, resulting in hypertriglyceridemia, lactic acidosis, and hyperuricemia. Other symptoms may occur in patients with Glycogen storage disease type 1b, especially after adolescence, including hepato-nephromegaly, hepatic adenomas, chronic renal failure, neutropenia and neutrophil dysfunction [3].

This study is the first attempt to comprehensively understand the molecular mechanism of G6P1 function and interaction with the lipid bilayer to predict the impact of the identified Tunisian mutations on G6PT1 structure and function.

## Patients and methods

### Ethics statement

Three Tunisian patients (MT, MB and SI) from three unrelated families (FI, FII, and FIII) were investigated based on clinical features and biochemical data. All studied patients presented hepatomegaly, hypertriglyceridemia, hypercholesterolemia, hyperlactatemia, hyperuricemia, neutrophilia. All investigated patients were offspring of consanguineous marriages between first cousins, originated from different three areas of Tunisia: Bizerte, Jendouba and Medenine. Clinical features are reported in Table 1.

**Table 1:** Clinical, biochemical and molecular profiles of Tunisian patients with GSD 1b

Families	I	II	III
Patients	MT	BM	SA
Origin	Bizerte	Medenine	Jendouba
Gender	F	M	M
consanguinity	1st degree	1st degree	1st degree
Age (Years)	3	12	9
Age of onset (Months)	8	3	2
Age of diagnosis (months)	8	5	4
Hypoglycemia mmol/l VU : 5.5 mmol/L	2.09	1.1	0.55
Lactatemia mmol/L VU : 1.7 mmol/L	12.17	5.96	3.55
Neutropenia (/mm^3^) VU : 188–8000/mm^3^	1550	500	590
Hepatomegaly	+	+	+
Body heat	39	Nl, 37	Nl, 37
Growth retardation	+	+	+
Genotype	p.R300H/ p.R300H	p.R300H/ p.R300H	p.W393X/ p.W393X

M: male; F: female; Nl: normal

This study was approved by the Ethics Committee of the La Rabta Hospital in Tunisia since 2010, and the families provided informed consents prior to collecting blood samples. All procedures were in accordance with the ethical standards of the responsible committee on human experimentation (institutional and national) and with the Helsinki Declaration of 1975, as revised in 2000 and approved by the Ethics Committees of the respective Tunisian hospitals.

**Fig. 1:**
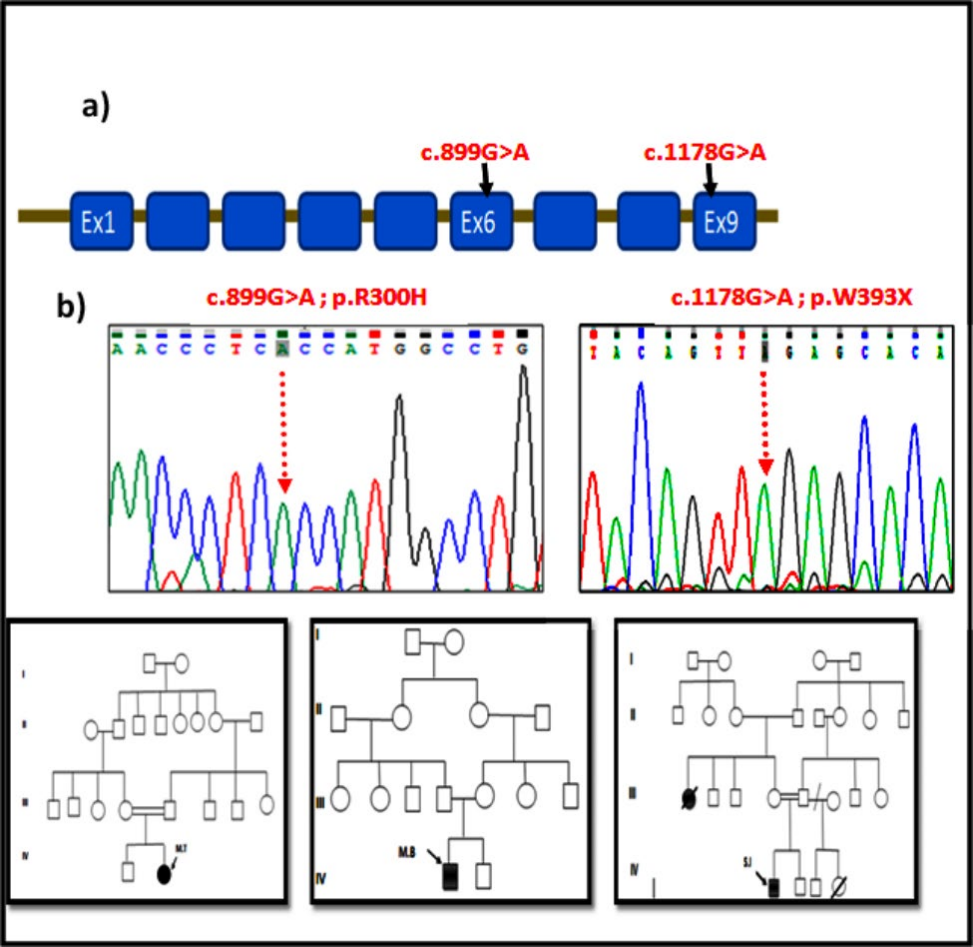
Family pedigree and electropherograms showing the reported mutation p.R300H and p.W393X associated with Glycogen storage disease type 1b **(a)** The right and left panel indicate the sequence electropherogram of the SLC37A4 mutations identified in exon 6 and 9 in the patients with GSD1b, respectively. **(b)** The right, the middle and the left panel concern the family pedigree of the patient MT, MB, and SI, respectively

Family I/Patient MT:

This four years old girl, resulting from a 1st degree consanguineous marriage (Fig. 1), originated from El Métouia and Ras El jbal North East Tunisia. She was 4 years old. This patient was referred to the children’s hospital of Tunis for suspected metabolic disease in 2016.

During the first year age, the patient presented with a fever of 39 °C accompanied by cough and clear rhinorrhea. At the age of a year and half, the evolution of the patient’s condition prompted her parents to consult to the Bab Saadoun children’s hospital in Tunis, leading the pediatrician to request the following assessment: blood count, CRP, procalcitonin, IgA weight assay, celiac serology, chest and abdominal X-ray and a thyroid assessment.

The performed abdominal ultrasound showed hepatomegaly and nephromegaly. Her faced presented with an aspect of hepatorenal overload suggesting the diagnosis of nephroblastoma, which is not confirmed because of the tumor lysis assessment. The glycemic cycle showed hypoglycaemia.

Her clinical data: hepatomegaly, chubby facies and severe hypoglycaemia after a short fast and neutropenia supported GSD 1b diagnosis.

Family II/Patient MB:

This five-month-old consanguineous baby originated from the town of Jendouba North West Tunisia (Fig. 1). He was admitted to pediatric hospital with exacerbation of respiratory illness, dyspnea at 60 breaths per minute, and then was transferred to the ICU for a push load. He was hospitalized for further investigation and nutritional rehabilitation. This patient was not pyretic but suffered from shortness of breath, cyanosis and refusal to breastfeed. During this period, the patient developed severe acidosis, severe hypoglycemia, hyperlactatemia, and hepatomegaly. This clinical feature suggested GSD1b.

Family III/Patient SI:

This five-month old consanguineous baby was born in Jerjisse South East Tunisia (Fig. 1) and hospitalized on suspicion of GSD1b. His medical history dates back to 2010. At the age of 3 months, the patient showed the following clinical symptoms: flatulence, watery diarrhea > 8 times / day but was apyretic and accepted to be breastfeed.

At 4 months of age, the patient was admitted to Ariana Regional Hospital and physical examination revealed abdominal distension, hepatomegaly, mild cytolysis, elevated cholesterol at 7.81 mmol / L, and hypoglycemia at 1.39 mmol / L. Then hypoglycemic levels ranged from 1.1 to 2.31 mmol / L and fasting blood glucosemia level was 1.44 mmol / L. His clinical feature and biological data looked the glycogen storage disease (GSD).

## Methods

### Molecular analysis

Human genomic DNA was isolated from peripheral leukocytes using a salting out method [6].

The DNA was used as a template for PCR amplification of the *SLC37A4* gene. The PCR amplification of the nine exons and intron-exon boundaries of the *SLC37A4* gene was carried as previously described. Sequencing was performed at the Laboratory of Biochemistry and Molecular Biology at the Béchir Hamza Children’s Hospital, Tunis as previously described [7, 8] using the forward (F; GTGGTGGGATTGCCAAAGAC) and reverse primer (R; AGGGTATCTGAGAGGCGAAG) for exon 6 and (F; TGTGTTGGGGAGTGGAAGGA) and (R; GGCGCAGAAATGGAAAGTGA) for exon 8 and 9.

### Molecular modeling

The computer-generated 3D structure model of the glucose-6-phosphate transporter 1 exchanger SLC37A4 was constructed with the protein homology modeling server SWISS-MODEL using the protein sequence retrieved from UniProt (UniProtKB id: O43826; https://www.uniprot.org/). The constructed tertiary structure was analyzed by DeepView Swiss-PdbViewer 4.1 [9] and POV-Ray 3.6 software. Further Crystallographic structure analysis and molecular dynamics simulation studies were performed to predict the impacts of this variant on protein stability using DynaMut tool [10] for the determination of the change in Gibbs free energy (∆∆G) and CABS-flex 2.0 server [11] (http://biocomp.chem.uw.edu.pl/) for fast simulations of protein structure flexibility upon mutation.

## Results

### Clinical and biochemical finding

Diagnosis was established by clinical presentation and measuring the biochemical parametres: glycemia, triglycemia, cholesterol, lactamia, uricemia, neutrophil blood cell count, inclouding neutrophil cell count.

Table 1 shows significant differences in these clinical and biological of parameters indicating phenotype heterogeneity between these GSD1b patients even though all patients presented hepatomegaly. Consanguinity was detected in all cases.

Hematological parameters were significant different in the studied patients. Neutropenia status was significantly different form moderate (patients MT and SI), severe (patient MB).

In addition the biochemical parameters showed a hypopglycemia for all patients, hyperlactatemia for patient SI, and hypercholesterolemia for patient MB. All patients had recurrent infections including otitis, respiratory tract infection.

Besides, the only patient MT had abdominal distention associated with watery diarrhea more than 8 times a day, the patient MB presented with abdominal distention.

### Molecular finding

Sequence analysis of the *SLC37A4* gene revealed two different mutations in the studied patients: two patients (MT and MB) from the families I and II were homozygous for p.R300H mutation. The patient SI from the third family was homozygous for the p.W393X mutation (Fig. 1).

### Bioinformatic finding

**Fig. 2:**
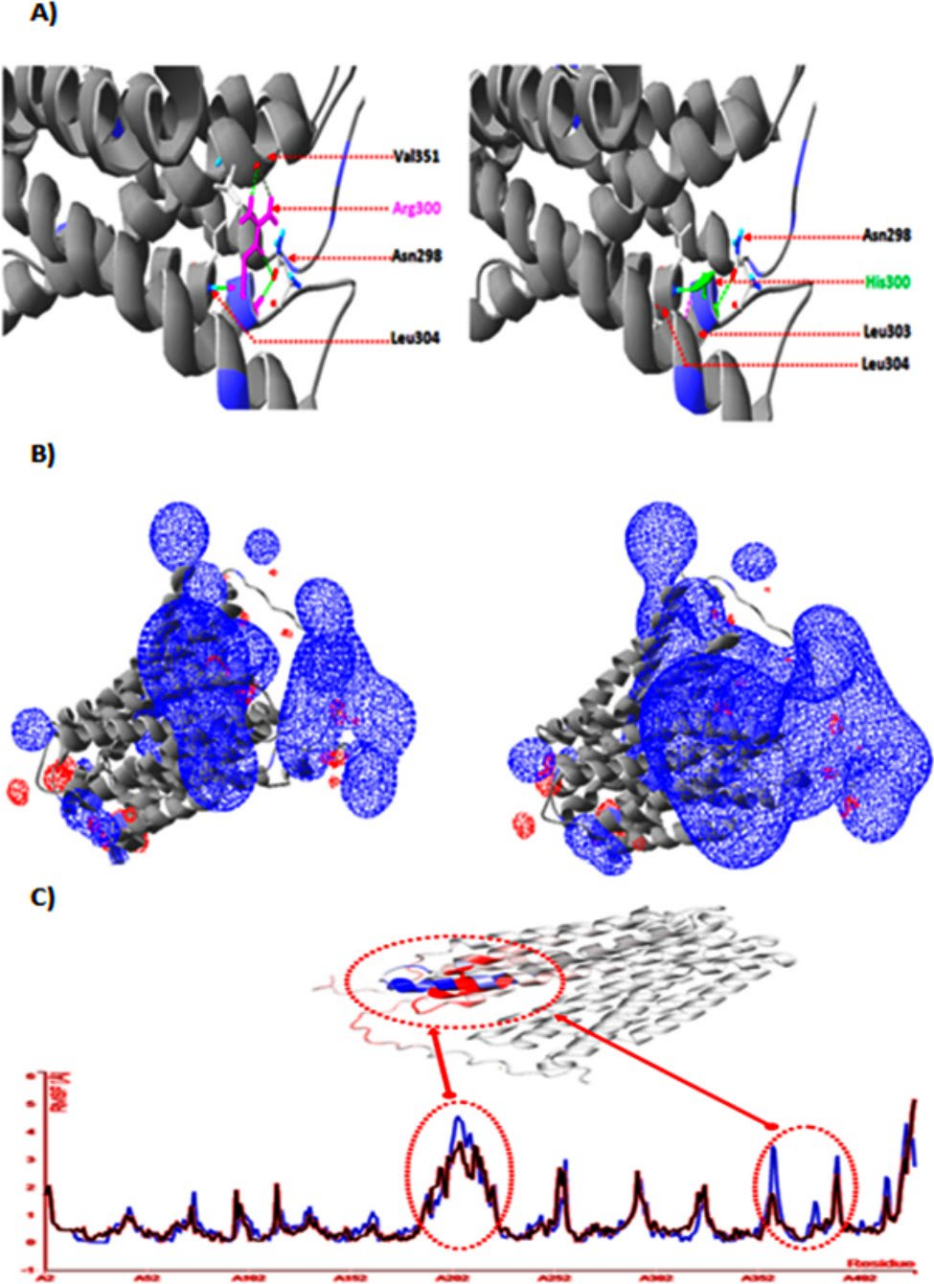
The Crystallographic structure analysis of the generated G6PT 3D structure model **A)** The analysis of the interactomic interaction before (left panel) and after mutations (right panel) introduction is indicated in the upper row. Wild-type Arg300 and mutant residues His300 are indicated dotted arrow and colored in pink and green, respectively. They showed as sticks alongside with the surrounding amino acids which are implicated on any type of interactions. **B)** The analysis of Electrostatic potentials before (left panel) and after mutations introduction (right panel) is shown in the middle and bottom rows, respectively. In this work, Electrostatic potentials were calculated using ionic strengths corresponding to 0 mM ion concentration and εP = 4. The negative and positive electrostatic potentials are highlighted in red and blue, respectively. **C)** The variation on protein stability after mutation introduction using DynaMut is shown in the bottom row. The amino acids are colored according to the vibrational entropy change upon mutation. Blue represents a rigidification of the structure and red a gain in flexibility. Backbone RMSD per residue for the wild type (red) and mutated protein (blue) are indicated in red and blue, respectively

The crystallographic structure analysis of the generated G6PT1 3D structure model suggests that the reported mutation p.R300H is located in the cytosol, closed to the lipid bilayer polar region. This mutation would lead to substitution of an important positive charged amino acid into another one with a small size and a less charged side chain (Fig. 2). This difference in charge could disturb the ionic interaction existing with the wild-type residue. The difference in size may cause an empty space in the core of the protein which could affect the normal folding of the protein.

On the other hand, the analysis of the electrostatic potential of the generated model showed that the missense mutation p R300H introduces an important positive charge in the polar head region of the phospholipid bilayer membrane. Consequently, this mutation could affect the protein folding and stability and/or may disrupt the interaction with the hydrophilic head region of the lipid bilayer which is essential for the activity of the protein.

Furthermore, further 3D structure analyzes demonstrated that the wild-type residue Arg300 forms a hydrogen bond with Val351, Asn298, and Leu304. The difference in size between the wild-type and mutant residue clearly shows that the new residue His300 is not in the correct position to make the same hydrogen bond as the original wild-type residue does (Fig. 2).

The analysis of the protein structure flexibility upon mutation using DynaMut tool and CABS-flex 2.0 server showed that the reported p.R300H mutation increases the molecule flexibility of in the cytosol region. The new physico-chemical properties introduced by the p.R300H mutation prevent the normal folding of the transmembrane domain in the cytosol region and eventually may destabilize the conserved structure which is essential for the correct function of the protein (Fig. 2).

## Discussion

The high rate of consanguineous marriage in Tunisia suggests a high incidence of autosomal recessive disorders. Thus is the first Tunisian published study, presenting the confirmation of the GSD1-type b. In fact, the definitive diagnosis is based on measuring the enzymatic activity of G6PT1 on a non-frozen liver biopsy. This technique is not available in Tunisia.

### Phenotypic expression of GSD 1b

The clinical manifestations of glycogen storage disease type 1b are heterogeneous, most are healthy at birth and clinical symptoms appear gradually in varying degrees. The deficiency of the glucose 6-translocase T1 system generally appears around 4 to 6 months (sometimes earlier).

In the present study, the first clinical signs appear at an average age of 4 months with a time interval ranging from 2 months to 8 months, which is consistent with the data in the literature [9].

### Metabolic phenotype of GSD1b

In this study, the metabolic phenotype of GSD 1b patients was characterized by episodes of hypoglycemia. This main biological parameter of the disease causes seizures associated with lactic acidosis which clinically often results in hyperventilation; the latter is a parameter that parents can easily watch over [12].

In addition, the three patients (MB, MT and SI) have presented episodes of hypoglycemia ranging from 0.55mmol/l to 2.09mmol/l; besides, the reason for consultation of SI was respiratory distress, which is consistent with data from the literature [13].

The accumulation of glycogen in the liver is due to the impossibility of transforming it into glucose, this accumulation causes hepatomegaly which is manifested by abdominal distension. It is noteworthy to mention that this symptom represented the main reason for consultation in the most studied patients (for 83% of cases) with glycogen storage disease type 1b [14].

In this serie, all patients presented with hepatomegaly at diagnosis, but only the 2nd patient consulted mainly for abdominal distension. These data are in agreement with the literature [14]. In addition, an Egyptian study showed that 90% of patients with GSD 1b also had chubby facies [15]. In this study, only the first patient MB of the first family developed large, well-filled cheeks.

### Immunohematological phenotype of GSD1b

Neutropenia turned out to be specific to type 1b glycogen storage disease, which leads to cause recurrent infections and inflammatory bowel disease. Its mechanism is unknown and its absence does not exclude the diagnosis of glycogen storage disease type 1b [16].

In the present study, the studied patients developed differently the neutropenia; in fact, patient MB had mild neutropenia of 1550/mm3, while the two patients MT and SI presented moderate neutropenia of 500 and 590mm3 respectively. Furthermore, patient SI suffers with recurrent E. coli pyelonephritis-like infections.

Dietary management is essential in the treatment of patients with GSD 1b, in order to prevent hypoglycaemia and ensure satisfactory height and weight growth. In addition corn starch (maïzena®) therapy can be started at six months of age in children under 2 years at intervals of 3 to 3.5 h with a maximum interval of 4 to 5 h, once the pancreatic amylase activity was measured [17].

### Molecular phenotype of GSD1b

In the current study, molecular analysis showed two previously identified mutations in three unrelated families: the p.R300H missense mutation and the p.W393X nonsense mutation. All the the patients carried a homozygous SLC37A4 mutation due to the high parental consanguinity rate.

The instability and G6P transport activity of G6PT1 are due to the presence of different mutations in *SLC37A4* gene and its helical/non helical distribution, its transmembrane helices location and its cytoplasmic N- and C- terminal domains location.

## p.R300H mutation

The two unrelated patients (MT and MB) with variable phenotypes were found homozygous for the described missense mutation which is located in exon 6 of *SLC37A4* gene. In addition, both patients were offrespring from consanguineous marriages.

The molecular characterization of the parents is useful to verify the segregation of the alleles therefore all the patients were homozygous and their parents were heterozygous.

This missense mutation (c.899G > A; p.R300H), results in the substitution of a guanine (CGC) with an adenine (CAC) at position 899 of cDNA, leading to modification of an arginin by a histidine at position 300 of G6PT1 protein. This mutation has been reported for the first time in a patient who was compound heterozygous and presenting a severe phenotype of GSD1b [18]. In this report, the missense mutation p.R300H was identified in the studied patient (MB) in homozygous state, and no additional mutations were found in this patient.

Crystallographic structure analysis of the generated model carrying the p.R300H missense mutation demonstrated that the new physico-chemical properties introduced by the mutated residue could prevent the normal folding and fuction of the glucose-6-phosphate transporter.

It has been noted that in the case of a compound heterozygous patient, there is a close relationship between the residual activity retained by the patient’s G6PT1 protein and the susceptibility of the GSD 1b patient to neutropenia as well as myeloid dysfunctions [19]. However, in an homozygous patient the abolished microsomal G6P uptake activity leads neutropenia with variables degrees (MB and SI), suggesting the optimal role of other biochemical parameters in phenotypic expression.

## p.W393X mutation

Several studies showed that SLC37A4 nonsense mutations have been described in severe form of GSD1b [18]. The c.1178G > A in patient SI within severe form of GSD1b is located in the 9th loop of the protein and produces a transporter lacking the tenth helix and the entire cytoplasmic loop, resulting in a truncated, unstable and non-functional G6PT1 protein  [16, 19–21].

Interestingly, the translation terminating mutations give rise to premature stop codon and truncated polypeptides. They are described in 11% of all gene lesions causing human inherited disease leading to the degradation of the partially translated protein and its loss of function and they are usually associated with a reduction in the steady-state level of cytoplasmic Mrna [22].

## Conclusion

In summary, the phenotypic expression in patients with GSD1b is due to the association of biochemical, clinical and molecular data. The heterogeneity of the phenotype in homozygous patients with the same missense mutation (p.R300H) could be due to the presence of polymorphisms, or other extra-genetic lesions not yet identified. In order to develop this option, it is necessary to carry on investigation of the study population in order to better understand the impact of these mutations in the microsomal activity of the protein.

The mutation spectrum of the *SLC37A4* gene was used for prenatal diagnosis to prevent this inheritable condition in our country which is characterized by a high rate of consanguinity.
